# Diagnosis of Extrapulmonary Tuberculosis at the Interface of Pathogen Detection and Host Response: Molecular, Microbiological, and Histopathological Advances

**DOI:** 10.3390/pathogens15101075

**Published:** 2026-10-10

**Authors:** Qibin Liu, Yuhui Jiang

**Affiliations:** 1 Wuhan Pulmonary Hospital (Wuhan Institute for Tuberculosis Control), Hubei Branch of the National Clinical Research Center for Infectious Diseases, Wuhan 430030, China; liuqibin0221@163.com; 2 National Engineering Research Center for Nanomedicine, Huazhong University of Science and Technology, Wuhan 430074, China

**Keywords:** extrapulmonary tuberculosis, molecular diagnosis, Xpert MTB/RIF Ultra, metagenomic next-generation sequencing, tuberculous granuloma, histopathology, host biomarkers, diagnostic algorithm

## Abstract

Extrapulmonary tuberculosis (EPTB) accounts for approximately 16% of notified tuberculosis (TB) cases worldwide and disproportionately affects children and immunocompromised populations. Paucibacillary disease, anatomically diverse sites, and nonspecific presentations make EPTB one of the most persistent diagnostic challenges in TB control. This review integrates microbiological, molecular, sequencing-based, and histopathological approaches to EPTB diagnosis within the context of site-specific immunopathology, including granuloma biology and the microenvironment of caseous necrosis. Comparative evidence is synthesized across osteoarticular, lymph node, pleural, central nervous system, and disseminated EPTB, focusing on Xpert MTB/RIF and Ultra, targeted and metagenomic next-generation sequencing (mNGS), and emerging host-response biomarkers and AI-assisted histopathology. Pragmatic, tiered diagnostic algorithms are proposed for resource-limited settings, where most EPTB occurs, and unresolved gaps in current international guidance are identified. Aligning diagnostic modality choice with anatomical site, specimen quality, pathological subtype, and laboratory capacity can shorten the time to EPTB diagnosis and inform host-directed diagnostic strategies.

## 1. Introduction

Tuberculosis (TB) remains the leading cause of death from a single infectious agent. In 2024, an estimated 10.7 million people developed TB and 1.23 million died from it, while 8.3 million new episodes were notified to national authorities [1]. Approximately 16% of notified cases were extrapulmonary, and this proportion exceeds 20% in several high-burden countries, including India, Bangladesh, and Ethiopia [1]. EPTB encompasses a spectrum of anatomically distinct manifestations—lymph node, pleural, osteoarticular, central nervous system (CNS), abdominal, genitourinary, and disseminated forms—all characterized by a low and unevenly distributed bacillary burden. In the 2019 Global Burden of Disease analysis of 33 bacterial pathogens, five pathogens—*Staphylococcus aureus*, *Escherichia coli*, *Streptococcus pneumoniae*, *Klebsiella pneumoniae*, and *Pseudomonas aeruginosa*—were responsible for 54.9% of an estimated 7.7 million deaths associated with the 33 bacterial pathogens assessed; *M. tuberculosis* was not included in that analysis and was estimated separately to account for 1.21 million deaths in 2019 [2]; within the tuberculosis toll, EPTB contributes disproportionately to diagnostic delay, invasive work-ups, and irreversible sequelae such as paraplegia and hydrocephalus.

The diagnostic challenges of EPTB are rooted in structural rather than incidental factors. First, most EPTB lesions are paucibacillary: meta-analytic sensitivity of Xpert MTB/RIF (Cepheid, Sunnyvale, CA, USA) across extrapulmonary specimens ranges from roughly 50% in pleural fluid to about 85% in tissue and lymph node aspirates, consistently below sputum performance [3]. Second, the anatomical sites most commonly involved—the spine, the meninges, the pleural space, and deep lymph node chains—are least amenable to simple sampling. Third, clinical and radiological presentations overlap extensively with malignancy, fungal infection, and non-infectious inflammatory disease, so the decision to biopsy is often delayed; in a recent tertiary cohort, 43.5% of patients with extrapulmonary or disseminated TB required more than one invasive procedure to achieve microbiological confirmation, and diagnostic delay was significantly longer than for pulmonary TB [4]. Fourth, immune status reshapes both disease phenotype and test performance: children, people living with HIV (PLWH), and other immunocompromised hosts present with higher proportions of disseminated and smear-negative disease, and WHO-commissioned Cochrane reviews consistently report lower molecular test sensitivity in these groups [3,5].

Over the past five years, the diagnostic landscape has shifted in three directions that this review synthesizes. Molecular cartridge-based assays have been joined—and in tertiary settings complemented—by next-generation sequencing (NGS), both targeted (tNGS) and metagenomic (mNGS) [3,6,7,8]; host-response biomarkers, led by parsimonious blood transcriptomic signatures, have matured from discovery to prospective validation [9,10]; and AI has begun to digitize the oldest EPTB diagnostic modality, histopathology [11,12]. Because the immunopathology of the tuberculous granuloma determines where, how many, and in what physiological state bacilli are present at a given site, diagnostic strategy cannot be separated from granuloma biology. This review therefore (i) summarizes the immunopathological basis that governs detectability of *M. tuberculosis* across EPTB sites; (ii) compares conventional microbiological, molecular, sequencing-based, and histopathological methods; (iii) examines host biomarkers and AI-assisted pathology; (iv) proposes site-specific, resource-tiered diagnostic algorithms; and (v) identifies gaps in current guidance and priorities for research. Wherever the supporting evidence derives from pulmonary TB cohorts or from experimental models rather than from EPTB-specific clinical studies, this is now identified explicitly in the text, and the limits of extrapolation across biological matrices and anatomical sites are discussed.

## 2. The Immunopathological Basis of EPTB Diagnosis

### 2.1. Granuloma Architecture and Bacillary Distribution

The tuberculous granuloma is the structural unit of EPTB pathology and simultaneously the principal obstacle to its laboratory diagnosis. Granulomas range from cellular, non-necrotizing aggregates—in which bacilli reside intracellularly within activated macrophages—to caseating lesions in which a central acellular zone of caseous necrosis is rimmed by epithelioid macrophages, multinucleated giant cells, and a lymphocytic cuff [13]. Caseation results from a convergence of immune-mediated macrophage death, ischemia, and hypoxia. In pulmonary and animal granulomas, the necrotic core is markedly hypoxic and enriched in hypoxia-inducible factor 1-alpha (HIF-1α), yet it is frequently sterile or nearly so; the highest viable bacillary burdens are typically found in the less hypoxic, lipid-rich peripheral rim, within foamy macrophages [13].. This rim–core gradient is an average tendency rather than a fixed rule: multimodal single-cell profiling of macaque lung granulomas has shown that cellular composition, bacterial burden, and local immune states vary dramatically even between adjacent lesions, with individual lesions ranging from rim-dominated to core-dominated or more even distributions [14]. Two caveats therefore qualify this spatial model. First, the most direct evidence for rim-peripheral viable bacilli and a frequently sterile caseous core derives from pulmonary and animal granulomas [13,14]; quantitative data from the extrapulmonary compartments addressed by the algorithms in Section 8 are considerably sparser, so the sampling implications below represent a biologically grounded extrapolation rather than a site-by-site validated rule. Second, bacillary distribution varies between lesions, and rim-first sampling will not be achievable in every specimen (e.g., fibrotic lymph nodes or sinus-tract material). This spatial heterogeneity has direct diagnostic consequences: a biopsy that samples only the necrotic core may yield abundant suggestive histology but few viable or intact bacilli, depressing culture yield and, to a lesser degree, nucleic acid detection. Wherever feasible, therefore, biopsies should target cellular, viable-appearing portions of a lesion—preferentially the granuloma rim or the abscess–tissue interface—rather than necrotic debris alone, while recognizing that this principle is extrapolated and should be tested prospectively in site-specific EPTB cohorts.

### 2.2. Hypoxia, Dormancy, and the Limits of Culture

The hypoxic, acidic, nitric-oxide-rich caseum drives *M. tuberculosis* into a non-replicating, drug-tolerant state governed by the dormancy survival regulon (DosR) [13]. Dormant bacilli may remain metabolically intact and DNA-positive yet fail to grow in culture—one of several non-exclusive contributors to the discordance between strongly positive molecular tests and negative cultures in caseous EPTB lesions. Such discordance is multifactorial: it may equally reflect a bacillary burden near the limit of detection, non-viable or residual DNA from killed organisms, losses during decontamination and specimen processing, prior anti-tuberculosis treatment, and other pre-analytical factors [15,16,17]; dormancy should therefore be regarded as a plausible partial explanation, supported by experimental data, rather than as a general or categorical one. Site-specific biology compounds this effect: cerebrospinal fluid (CSF) is both low-burden and bacteriostatic; pleural fluid contains bacilli in only a minority of immunologically confirmed cases; and bone lesions are frequently sampled through sinus tracts contaminated by skin flora. Granuloma physiology thus accounts for three empirical patterns observed throughout this review: (i) molecular assays generally outperform culture in paucibacillary EPTB; (ii) sensitivity is specimen-site-dependent rather than assay-fixed; and (iii) histopathology retains value precisely where organisms are undetectable because the granulomatous pattern itself reflects the host response to the pathogen.

### 2.3. Site-Specific Immune Phenotypes and Their Diagnostic Signatures

**Figure 1:**
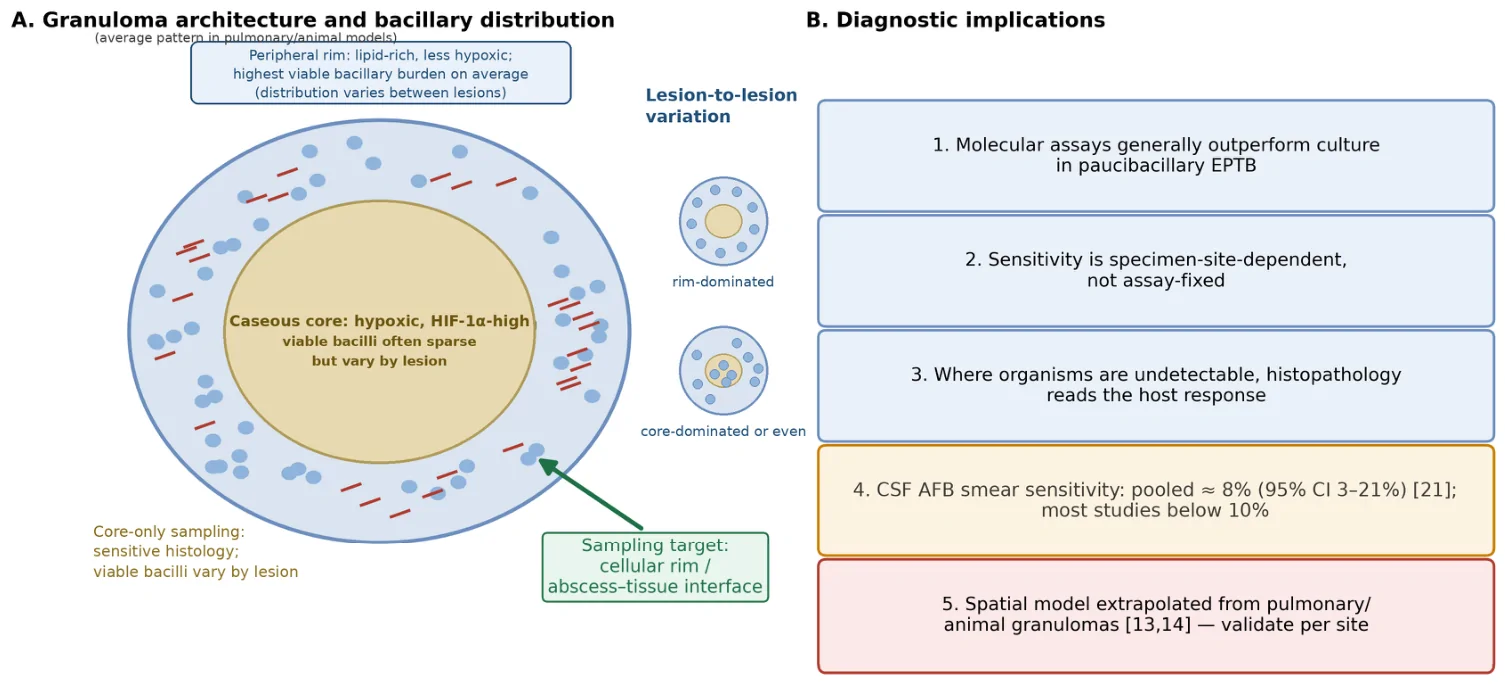
Granuloma architecture as a determinant of diagnostic detectability in extrapulmonary tuberculosis. (**A**) Schematic granuloma summarizing the average pattern described in pulmonary and animal models: viable bacilli tend to concentrate in the lipid-rich, less hypoxic peripheral rim, whereas the hypoxic, HIF-1α-high caseous core is frequently sterile or nearly so. Because bacillary distribution varies considerably between individual lesions (two contrasting examples are sketched to the right of the granuloma), the rim–core gradient should be read as a tendency rather than as a fixed rule. (**B**) Diagnostic implications for sampling and assay choice. HIF-1α, hypoxia-inducible factor 1-alpha; AFB, acid-fast bacilli. Note: the spatial model is derived predominantly from pulmonary and animal granulomas [13,14]; its generalization to extrapulmonary sites should be read as a biologically grounded extrapolation pending site-specific validation. CSF AFB smear sensitivity in adults with tuberculous meningitis is very low (pooled 8%, 95% CI 3–21%), with most individual studies reporting values below 10% [21].

The immune response varies by anatomical compartment, and with it the pattern of biomarker expression. Tuberculous meningitis (TBM) induces a compartmentalized CSF immune response with interferon-γ (IFN-γ)-inducible chemokines, raised proteins, and hypoglycorrhachia [18,19]. Pleural TB is driven by a local T-helper-1 response in which adenosine deaminase (ADA) and IFN-γ in pleural fluid reflect recruited antigen-specific T cells—useful adjuncts precisely because the fluid itself so often contains no detectable bacilli. Lymph node TB occupies an intermediate position, with higher bacillary loads than CSF or pleura, particularly in HIV-associated disease [20]. These site-specific immune signatures are not incidental: they are the biological substrate for the host biomarkers discussed in Section 7 and for the combined microbiology-plus-biomarker algorithms proposed in Section 8 (Figure 1).

## 3. Conventional Microbiological Methods

### 3.1. Smear Microscopy

Acid-fast smear microscopy is rapid, inexpensive, and universally available, but its sensitivity in EPTB is poor—commonly 20–50% for tissue specimens [3,22] and very low in CSF. In adults with TBM, a systematic review and diagnostic test accuracy meta-analysis found a very low pooled sensitivity of CSF acid-fast bacilli (AFB) smear (8%, 95% CI 3–21%), with most individual studies reporting values below 10% [21]. Microscopy retains a niche role in high-burden settings for lymph node aspirates and abscess material, but a negative smear never excludes EPTB (Table 1).

### 3.2. Culture

Mycobacterial growth indicator tube, MGIT; Becton Dickinson, Franklin Lakes, NJ, USA)—remains Mycobacterial culture—solid (Löwenstein–Jensen)—remains the reference standard and the indispensable gateway to phenotypic drug-susceptibility testing (DST). Its weaknesses in EPTB are well known: turnaround times of 2–8 weeks, sensitivity ceilings in paucibacillary specimens (roughly 30–70% depending on site and processing) [3,4], and vulnerability to decontamination-induced loss of stressed bacilli. In the German tertiary cohort of Wilmink et al., culture positivity was 71.8% on native tissue, but only when specimens were kept unfixed; formalin fixation, common when TB is not suspected at first biopsy, destroyed culture potential and forced repeat procedures in a large fraction of patients [4]. The operational lesson, applicable everywhere, is that every tissue specimen from a suspected EPTB site should be split at collection for histology (formalin), microbiology (native, sterile container), and molecular testing. A consolidated performance comparison of all modalities discussed in Section 3, Section 4, Section 5, Section 6 and Section 7 is provided in Table 1, in which each estimate is now cited to its source with the corresponding reference standard and study population indicated.

## 4. Molecular Diagnostics: Xpert MTB/RIF and Ultra, Line-Probe Assays, and Emerging Platforms

### 4.1. Cartridge-Based Nucleic Acid Amplification

The WHO-endorsed Xpert MTB/RIF and its successor Xpert MTB/RIF Ultra are the backbone of rapid EPTB diagnosis [33]. Ultra’s redesigned multicopy IS6110/IS1081 targets and larger reaction chamber lowered the limit of detection from approximately 131 to 16 colony-forming units (CFU)/mL equivalent—a critical gain in paucibacillary disease [34,35]. The WHO-commissioned Cochrane meta-analysis in adults [3] and the companion review in children [5] provide the most reliable performance frame: Ultra consistently outperforms Xpert on smear-negative and extrapulmonary specimens, at the cost of slightly lower specificity and a trace-detected category that complicates rifampicin resistance calls [15,16]. A trace call indicates the presence of a very low amount of *M. tuberculosis* DNA and, in the appropriate clinical context, supports a diagnosis of TB; this should be distinguished from the separate problem that rifampicin resistance cannot be determined from a trace result and that trace calls require cautious, often phenotypic, confirmation [15,16,17]. Pooled analyses of Ultra versus Xpert concur on this sensitivity–specificity trade-off [36,37]. Real-world series confirm the pattern across settings: Ultra improved detection across lymph node, pleural, and other extrapulmonary specimens in Tunisia [38]; in lymphadenitis specifically, Ultra achieved high sensitivity even in a high-HIV setting [20]; and multi-site evaluations from the Middle East and North Africa report concordant gains [39]. Early clinical validation of Xpert on extrapulmonary specimens already demonstrated the site-dependence of sensitivity that still shapes guidelines today [22], and WHO rapid communications subsequently consolidated Ultra’s role as an initial rapid diagnostic for both pulmonary and extrapulmonary TB [40].

In tuberculous meningitis—the most lethal EPTB form—Ultra has improved detection but remains insufficiently sensitive to exclude TBM. A prospective randomized head-to-head study reported higher sensitivity point estimates for Ultra than for Xpert, although the difference was not statistically significant across definite, probable, and possible case-definition strata [23], accompanied by an analysis stressing that a negative Ultra still does not exclude TBM [41]. Pooled Xpert sensitivity in CSF varies with the reference standard, from about 42% against composite clinical reference standards [3] to 56–63% in pooled analyses using clinical or culture standards [26] and 81–85% when restricted to definite or probable TBM [25]; adult studies show sensitivity gains from CSF centrifugation [42] and a consistent direction of effect in direct comparative series [43]—a multicenter prospective study comparing Ultra with a cell-free nucleic acid assay further showed that assay ranking depends on the reference standard applied [44]. In children, the evidence is now firmer: an Indian multicenter study reported Ultra sensitivity of 50% versus 18% for Xpert [24], and a 2025 Bangladeshi study of 187 children found Ultra sensitivity of 88% (versus 34% for Xpert and 30% for culture) against the uniform clinical case definition, and 100% against a composite microbiological reference standard, detecting 26 cases missed by all other methods, most of them in the trace-detected category [27]. Because TBM treatment cannot await culture, current practice guidance recommends a central role for Ultra on CSF, ideally from centrifuged large-volume samples, integrated with the uniform clinical case definition [45] and adjunctive biomarkers [18,19].

### 4.2. Line-Probe Assays, Real-Time PCR, and Other NAATs

Line-probe assays (e.g., GenoType MTBDRplus; Bruker/Hain Lifescience, Nehren, Germany) and chip-based NAATs extend rapid molecular detection and first-line resistance profiling; genotypic resistance testing has been specifically evaluated on extrapulmonary specimens in high-burden settings [46]. The Truenat platform (Molbio Diagnostics, Goa, India) has been validated for EPTB in single-center and multi-center evaluations [47,48,49], droplet digital PCR (ddPCR; Bio-Rad, Hercules, CA, USA) remainthe CapitalBio Mycobacterium real-time PCR assay (CapitalBio Technology, Beijing, China) has performed comparably, reinforcing the decentralization trend. In-house real-time PCR and valuable where cartridges are unaffordable: in a multicenter evaluation, ddPCR detected MTB DNA in adult TBM CSF with higher sensitivity than Xpert on the same specimens [30]—a comparative finding that is specimen-, population-, and reference-standard-dependent and should not be generalized uncritically to other settings—and alternative commercial real-time PCR assays have performed comparably to Xpert on CSF [50]. A chip-based CSF Truenat assay showed feasibility for TBM at tertiary centers [51]. Wherever NAATs are used on tissue, formalin-fixed specimens remain testable—an important fallback when TB was not suspected at first biopsy [4]—but NAAT performance on formalin-fixed, paraffin-embedded (FFPE) material is strongly influenced by fixation duration and DNA preservation, so negative results on poorly preserved tissue do not exclude EPTB, and very low trace calls require cautious interpretation [17].

### 4.3. CRISPR-Based Detection and Cell-Free DNA

Among the emerging platforms, CRISPR-based diagnostics are the closest to clinical translation. Proof-of-concept Cas12 and Cas13 assays targeting IS6110 achieved sensitivities of 88–95% with sub-2 h workflows at costs below cartridge-based systems [52,53,54], and clinical evaluation of CRISPR-Cas13a on clinical specimens has followed [55]. Beyond DNA, genome-wide tiled Cas13 detection of circulating *M. tuberculosis* cell-free DNA offers a sputum-independent, site-independent approach with potential for disseminated and deep-seated EPTB [56]. Reviews of the field converge on sub-2 h, equipment-minimal workflows as achievable, while noting that standardization and prospective accuracy studies against clinical reference standards are still required before endorsement [57,58,59,60]. None of these platforms is yet WHO-endorsed for EPTB; they remain at the proof-of-concept to early clinical-validation stage, and their incorporation into practical diagnostic algorithms should therefore be clearly distinguished from established approaches (Section 8); they will require further clinical validation, standardization, implementation evidence, and regulatory or guideline endorsement before routine adoption. Nonetheless, these platforms are likely to shape the next generation of point-of-care molecular tests.

## 5. Sequencing-Based Approaches: tNGS and Metagenomic NGS

### 5.1. Metagenomic NGS

NGS-based diagnostics address the two failure modes of targeted NAATs in EPTB: the requirement for a priori target knowledge and the inability to detect non-tuberculous mycobacteria, fungi, or mixed infections that mimic EPTB clinically and histologically. In CNS disease, two meta-analyses consistently found mNGS sensitivity in definite TBM of roughly 60–80% with specificity near 100%, outperforming smear, culture, and—in several cohorts—Xpert [6,31]. Early CSF cohorts in HIV-negative patients reported sensitivity gains over conventional methods [60], and a 2024 study again found mNGS sensitivity in definite TBM of 78.6%, versus 0% for smear and culture and 14.3% for Xpert on the same CSF aliquots [32]. A dedicated review of CSF mNGS for the rapid diagnosis of tuberculous meningitis reaches consistent conclusions [61].

Outside the CNS, a 2024 retrospective analysis of 162 suspected osteoarticular TB cases reported mNGS sensitivity of 85.1% and specificity of 100% on abscess and tissue specimens—similar to Xpert (95.7%) and superior to MGIT culture (68.1%) [7]. In cervical lymph node TB, a 2025 study of ultrasound-guided puncture biopsies found mNGS sensitivity of 89.5%, significantly higher than culture (43.3%) and Xpert (68.4%), with the added benefit of detecting mixed bacterial, fungal, and viral co-infections and of remaining reliable after prior anti-TB therapy [8]. It should be emphasized that the 100% specificity figures reported in these and the CSF cohorts are findings of individual studies—often with modest non-TB control groups—rather than general test characteristics. These findings justify a clear operational positioning: mNGS serves as a second-line, high-yield reflex test for EPTB where first-line microbiology is negative, and pre-test probability remains substantial—particularly for deep-seated or partially treated disease—rather than a universal first-line screen, given cost, turnaround time (24–72 h), dependence on nucleic-acid extraction quality, and interpretation pitfalls such as low read counts and environmental mycobacteria.

### 5.2. Targeted NGS

Targeted NGS (tNGS) encompasses two distinct applications that are frequently conflated and are best considered separately. The first is broad-range, multiplex PCR-based panels that enrich dozens to hundreds of pathogen targets to identify the etiologic agent in tissue or fluid—conceptually intermediate between mNGS breadth and NAAT simplicity, with lower cost and faster turnaround; robust EPTB-specific accuracy data for such broad-range tNGS panels remain limited. The second is targeted *M. tuberculosis* sequencing (amplicon- or capture-based), whose primary purpose is genotypic drug-resistance prediction; this application has been endorsed by WHO for the rapid detection of drug-resistant TB from cultured isolates or validated direct specimens [62], but it addresses a different question (resistance profiling) from EPTB diagnosis, and its accuracy as a primary EPTB diagnostic remains insufficiently established. For either application, standardization of enrichment panels, bioinformatic thresholds, and reporting criteria is an open need. For the near term, tNGS is best positioned where resistance prediction is urgent (e.g., previously treated osteoarticular or disseminated TB) and culture-based phenotypic DST is too slow.

## 6. Histopathology and AI-Assisted Pathology

### 6.1. The Enduring Role of Histopathology

Histopathology remains the most widely available and frequently decisive supportive EPTB diagnostic modality in resource-limited settings, where most EPTB occurs. Necrotizing granulomas with caseation are highly suggestive, though not pathognomonic—sarcoidosis, fungal infection, and malignancy must be excluded—and the presence of AFB on tissue sections strengthens specificity [4,13]. Because necrotizing granulomas are not specific for TB, histopathological evidence should be integrated with microbiological confirmation whenever the latter is obtainable, and alternative infectious (non-tuberculous mycobacteria, fungi) and non-infectious (sarcoidosis, malignancy, vasculitis) causes must be actively excluded [4,13]. Histology also has a unique operational advantage: formalin-fixed, paraffin-embedded material remains available for retrospective NAAT even when native specimens were never collected [4]. The diagnostic value of histopathology is greatest precisely in the paucibacillary situation, where it reads the host response when the pathogen itself is undetectable (Figure 1).

### 6.2. AI-Assisted Histopathology

Deep-learning systems now detect AFB and mycobacteria in Ziehl–Neelsen-stained tissue and whole-slide images, with reported overall accuracies of 86–96% [11,12]. A clinical study showed that AI-supported whole-slide pathology detected mycobacteria in transbronchial lung biopsies missed by bacteriology, with pathologist-level specificity [11]. A 2024 systematic review of computer-vision applications on digitized human lung tissue concluded that sensitivity and specificity above 90% are achievable, while highlighting the absence of shared datasets, model generalizability, and formal external validation as barriers to clinical deployment [12]. Two caveats are essential. First, nearly all available evidence derives from pulmonary tissue; application to EPTB—where tissue is often the only material—is an explicit extrapolation that requires site-specific validation before diagnostic claims are made. Second, overall accuracy alone poorly characterizes diagnostic performance; the sensitivity, specificity, validation setting, and dataset characteristics of the underlying studies (above-90% sensitivity and specificity in curated lung-tissue datasets, without external or EPTB-site validation) should guide interpretation. Accordingly, AI-assisted screening of granulomatous biopsies for AFB and necrosis patterns is best regarded as a promising, potentially low-infrastructure future application in high-burden, pathologist-scarce settings—rather than an established or near-term routine diagnostic approach.

## 7. Host-Response Biomarkers

Interferon-γ release assays (IGRAs) support—never establish—EPTB diagnosis by demonstrating sensitization, and their utility is mainly adjunctive, being relatively greater in children and in supporting the differentiation of TB from sarcoidosis in granulomatous disease than in confirming EPTB at most other sites. More substantive progress has come from blood transcriptomics. It must be emphasized that this evidence derives almost entirely from pulmonary TB cohorts; its application to EPTB is an explicit extrapolation that requires EPTB-specific validation. RISK6, a six-gene signature measurable by RT-qPCR on finger-prick blood, was validated across African cohorts—predominantly in pulmonary disease—for diagnosis, short-term progression risk, and treatment-response monitoring [9]. A 2022 prospective multicenter head-to-head validation of eight parsimonious signatures on a microfluidic RT-qPCR platform showed that several signatures approached or met WHO target product profile benchmarks for triage testing in symptomatic adults, while performance fell short in asymptomatic and HIV-positive subgroups [10]. A 2026 state-of-the-art review in this journal consolidates the translational status of RISK6 and related signatures, emphasizing population-dependent thresholds and interference from non-TB inflammation as the principal barriers [63], benchmarked against the WHO target product profiles for TB diagnostics [64].

For EPTB specifically, soluble and transcriptomic biomarkers remain less validated than for pulmonary disease—an explicit research priority, since a sputum-independent test would disproportionately benefit paucibacillary, hard-to-sample disease. Adjunctive lesion- and fluid-based markers are more mature in selected compartments: aptamer-based detection of mannose-capped lipoarabinomannan in lesion tissue has shown promise [65], and CSF biomarker panels (lactate, immunoglobulins, metabolomic profiles) are actively being evaluated for TBM diagnosis and prognosis [66,67,68]. The near-term realistic role of host biomarkers in EPTB algorithms is as triage and adjunctive evidence layered onto microbiological testing, not as stand-alone diagnosis.

## 8. Site-Specific Diagnostic Algorithms

**Figure 2:**
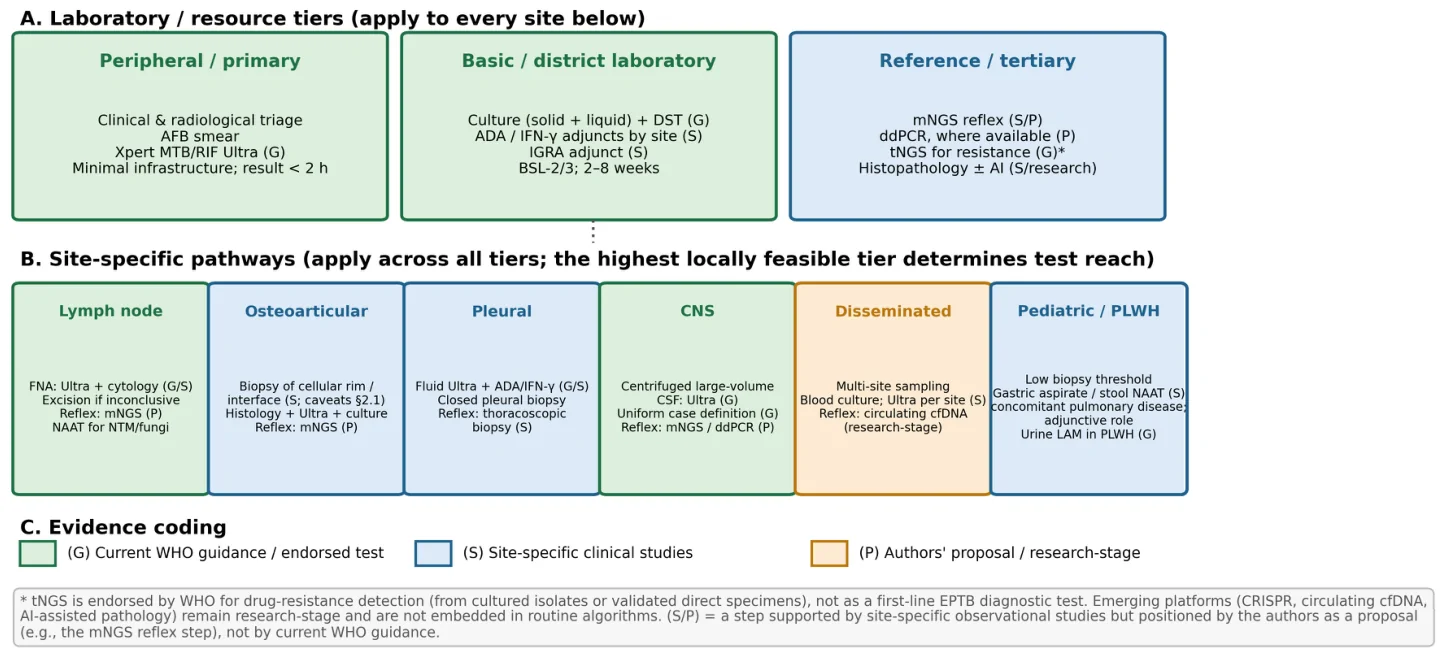
Tiered, site-specific diagnostic algorithm for extrapulmonary tuberculosis. (**A**) Laboratory/resource tiers are shown as a separate axis from (**B**) site-specific diagnostic pathways, so that disease sites are no longer placed beneath particular resource tiers; site pathways apply across all tiers, with the locally highest feasible tier determining which tests are reachable. (**C**) Evidence coding: green = current WHO guidance; blue = supported by site-specific clinical studies; orange = authors’ proposals or research-stage approaches pending prospective validation and endorsement. (S/P) indicates that a step is supported by site-specific observational studies but is positioned as proposed by the authors—for example, the mNGS reflex step—not by current WHO guidance. The orange shading of the Disseminated and Pediatric/PLWH pathways denotes that each pathway as a whole is positioned as a proposal or research-stage construct; individual steps within these pathways that carry a (G) label, such as urine LAM in PLWH, remain guideline-endorsed. Ultra, Xpert MTB/RIF Ultra; NAAT, nucleic acid amplification test; DST, drug-susceptibility testing; ADA, adenosine deaminase; IGRA, interferon-γ release assay; LAM, lipoarabinomannan; mNGS, metagenomic next-generation sequencing; ddPCR, droplet digital PCR; tNGS, targeted next-generation sequencing; cfDNA, cell-free DNA; AI, artificial intelligence.

The evidence base above converges on a small set of practical algorithms (Table 2; Figure 2). The unifying principle is tiered escalation: a rapid molecular anchor available at all levels, site-appropriate adjuncts at basic laboratories, and high-yield reflex testing at reference centers, so that a patient with persistent high pre-test probability is not left undiagnosed after a single negative test. To make the evidence status of each step transparent, algorithm components are labeled (G) where they reflect current WHO guidance, (S) where they are supported by site-specific clinical studies, and (P) where they represent the authors’ proposals whose positioning awaits prospective validation. In particular, mNGS-based reflex steps are supported by observational diagnostic-accuracy studies and are not part of current WHO-endorsed algorithms; they are presented as evidence-based proposals rather than established practice.

Feasibility considerations underpin this tiering. Smear microscopy and cartridge-based NAATs require minimal-to-moderate infrastructure and are already embedded in most national programs; culture requires biosafety level 2–3 containment and 2–8 weeks of turnaround but remains indispensable for DST; mNGS and ddPCR require nucleic-acid extraction capacity, dedicated instrumentation, bioinformatics or droplet-analysis support, and per-test costs that remain high relative to programmatic budgets in many high-burden countries, with turnaround times of 1–3 days, and, because many routine laboratories run these assays in batches, even a 24–72 h analytical turnaround may translate into days to weeks of actual reporting delay. Because published cost-effectiveness analyses of EPTB diagnostic algorithms remain scarce, it is necessary that this comparison be qualitative rather than a formal economic evaluation. We therefore position mNGS and ddPCR at the referral tier as reflex tests rather than routine first-line steps, and we confine emerging platforms (CRISPR, circulating cell-free DNA, AI-assisted pathology) to explicitly research-stage contexts until standardization, prospective clinical validation, and guideline endorsement are achieved.

Lymph Node TB: Fine-needle aspiration for Xpert Ultra plus cytology is the pragmatic first line (G/S); excisional biopsy is recommended when aspiration is inconclusive. Ultra is preferred over Xpert [3,20]; mNGS on biopsy tissue adds yield after partial treatment or when non-tuberculous mycobacteria or fungal mimics are plausible (P) [8].

Osteoarticular TB: Image-guided sampling that targets viable-appearing, cellular portions of the lesion—preferentially the abscess–tissue interface—rather than necrotic debris alone, with the extrapolation caveats of Section 2.1; submit for histology, Xpert Ultra, culture, and—if first-line tests are negative and suspicion high—mNGS, which matched or exceeded culture yield in the largest series (S/P) [7].

Pleural TB: No single test suffices: Xpert Ultra on pleural fluid has only moderate sensitivity, so fluid ADA and/or IFN-γ, pleural biopsy for histology and NAAT, and biopsy culture should be combined; closed or thoracoscopic pleural biopsy is the highest-yield step when less invasive tests conflict (G/S) [3,28,69].

CNS TB: Ultra on centrifuged, large-volume CSF as the rapid anchor (G), integrated with the uniform clinical case definition [45] and adjunctive CSF biomarkers [66,67,68]; mNGS as a reflex test when Ultra is negative, and suspicion remains high (P) [6,32]; ddPCR where available (P) [30]. Treatment must not await culture in probable or definite TBM [18,19].

Disseminated Disease: Multiple-site sampling, mycobacterial blood culture, and emerging circulating MTB cell-free DNA detection (research-stage) [56].

Abdominal and Genitourinary TB: Although named among the common EPTB manifestations in the Introduction, these sites are not assigned dedicated algorithms here because site-specific diagnostic-accuracy evidence remains comparatively sparse. The same principles apply: image-guided or endoscopic sampling of the most accessible lesion, split specimens for histology, native microbiology, and molecular testing, with NAAT plus culture. The algorithms presented should therefore be read as addressing selected, evidence-richest EPTB presentations rather than the full anatomical spectrum.

## 9. Special Populations and Resource-Limited Settings

Children carry a disproportionate EPTB burden, cannot expectorate, and progress faster. The original and updated pediatric Cochrane reviews document steady gains in non-sputum molecular testing [5,70], and a 2025 systematic review and meta-analysis found that stool-based molecular tests, with appropriate processing, approach the sensitivity of induced sputum—an operational advance for paucibacillary pediatric disease [71]—while detecting mainly concomitant pulmonary, often subclinical, disease rather than disease at the extrapulmonary site; however, gastric aspirate and clinical algorithms remain the fallback in most programs, as summarized in recent pediatric diagnostics syntheses [72]. CNS tuberculosis carries the heaviest toll across the lifespan: a global systematic review and meta-analysis quantified substantial morbidity and mortality from CNS TB [73]; outcomes hinge on pre-treatment diagnostic probability as much as on any single test [74]; and delayed recognition of tuberculous meningitis leads to encephalitic extension and irreversible sequelae [19].

In PLWH, dissemination and atypical histology lower test sensitivity across modalities [3,5]. Urine-based strategies—Xpert Ultra on urine and lateral-flow lipoarabinomannan assays (Determine TB-LAM; Abbott, Chicago, IL, USA)—were designed for pulmonary TB in advanced immunosuppression but have clear relevance to disseminated EPTB, with prospective evidence of diagnostic yield [75,76,77]. At the population level, EPTB proportions rise with HIV prevalence and diagnostic capacity; retrospective cohorts from sub-Saharan Africa and Asia consistently show lymph node and pleural predominance, diagnostic delay, and under-ascertainment [78,79,80]. For resource-limited settings, the realistic near-term routine stack at peripheral and basic laboratories is: clinical and radiological triage; smear plus Xpert Ultra on every obtainable specimen; site-appropriate ADA/IGRA adjuncts; and culture (with phenotypic DST) at the microscopy-center or reference tier. At the referral tier, mNGS and molecular DST are theoretically desirable reflex options but are realistically deployable only where instrumentation, reagent supply chains, bioinformatics support, and per-test cost permit. This stack presumes existing cartridge-based testing and culture capacity; in many low-income settings, even ddPCR and mNGS pose substantial technical, financial, infrastructure, and implementation challenges, and they should be regarded as referral-level options rather than universally scalable components. Distinguishing theoretically desirable referral-level options from approaches realistically deployable within routine diagnostic networks is essential when adapting these algorithms locally; retrospective data from Guangxi, China, illustrate the scale of the EPTB burden and the predominance of pleural and skeletal disease in a high-burden Chinese setting [80], but a single-region experience of this kind should not be read as a universal template.

## 10. Gaps, Guideline Limitations, and Research Priorities

Five gaps dominate the current landscape (Table 3). First, WHO guidance endorses Xpert/Ultra and selected NAATs for most extrapulmonary specimen types [40], but neither mNGS nor tNGS is currently recommended as an EPTB diagnostic test pending standardized accuracy data—urgently needed in CSF, pleura, and bone. This gap in diagnostic guidance should not be conflated with the separate, established WHO recommendation on targeted NGS for the rapid detection of drug-resistant TB, which addresses resistance profiling from cultured isolates or validated direct specimens rather than the diagnosis of EPTB itself [62]. Second, EPTB-specific host biomarker validation lags far behind pulmonary TB [10,63]. Third, the trace-detected Ultra category needs harmonized rifampicin-resistance interpretation, especially in CSF [16,17]. Fourth, CRISPR and cell-free DNA platforms require prospective, reference-standard-compliant accuracy studies in EPTB populations [53,56,57]. Fifth, diagnostic algorithms must be costed and tested for clinical impact—not only accuracy—particularly against outcomes such as time-to-treatment and neurological sequelae.

## 11. Conclusions

EPTB diagnosis is now genuinely multimodal, and no single test suffices. A combination—anchored by granuloma-aware sampling, Xpert Ultra as the rapid molecular backbone, culture for DST, mNGS as the high-yield reflex, and site-appropriate biomarker adjuncts—can approach actionable certainty at selected anatomical sites when the recommended tiered pathway is fully implemented. The decisive variables are no longer only analytically sensitive but include sampling strategy, specimen routing, and pre-test probability. Embedding these choices in site-specific, resource-tiered algorithms, while validating host-directed and sequencing-based innovations in well-designed prospective studies, offers a realistic route to shortening the diagnostic odyssey of EPTB and, ultimately, to reducing the burden of this infection. Throughout this revision, statements grounded in pulmonary or experimental evidence have been explicitly qualified, and research-stage technologies have been clearly distinguished from approaches ready for routine diagnostic algorithms.

## Figures and Tables

**Table 1:** Comparative performance of major diagnostic modalities in extrapulmonary tuberculosis.

Modality	Specimen/Site	Sensitivity	Specificity	Turnaround	Cost/Access Tier
AFB smear	CSF	Very low (pooled ≈ 8%; 95% CI 3–21%) [21]	High (≥95%) [21]	<1 h	Minimal
AFB smear	Tissue/aspirates	Low (≈20–50%) [3,22] (a)	High	<1 h	Minimal
Culture (solid/liquid)	CSF	Low (≈30–35%) [23,24] (b)	High	2–8 weeks	Basic
Culture (native tissue)	Lymph node/bone	Moderate (≈68–87%) (c)	High	2–8 weeks	Basic
Xpert MTB/RIF	CSF	≈42–85% depending on reference standard [3,25,26] (d)	High (>95%)	≈2 h	Moderate
Xpert Ultra	CSF	≈50–88% [23,24,27] (e)	Specificity slightly lower than Xpert in pooled EPTB analyses [3]	≈2 h	Moderate
Xpert Ultra	Lymph node aspirate/tissue	High (≈85–90%, pooled) [3,28] (f)	High	≈2 h	Moderate
Xpert MTB/RIF	Pleural fluid	Low (≈19% composite; ≈50% culture) [3,28] (g)	High	≈2 h	Moderate
Xpert Ultra	Pleural fluid	Moderate (≈75% vs. culture; 4 studies; very low certainty) [3] (g)	High	≈2 h	Moderate
ADA/IFN-γ	Pleural fluid	High (ADA pooled 92%; specificity 90%) [29] (h)	Moderate	Hours	Minimal
ddPCR	CSF	Higher than Xpert in one multicenter adult TBM study [30] (i)	High	≈1 day	Reference
mNGS	CSF	≈60–80% (definite TBM) [6,31,32] (j)	≈100% in individual studies [6,31] (k)	24–72 h	Reference
mNGS	Osteoarticular tissue/abscess	85.1% (single 2024 series) [7] (l)	100% (same series) [7] (l)	24–72 h	Reference
mNGS	Lymph node tissue	89.5% (single 2025 series) [8] (m)	100%(same series) [8] (m)	24–72 h	Reference
IGRA	Blood	Supportive only	—	Hours	Moderate
Transcriptomic signature (RISK6)	Blood	AUC ≈ 0.9 in symptomatic adults; evidence from pulmonary-TB cohorts—no validated EPTB estimate [9,10] (n)	—	Hours	Research

Notes: reference standards and evidence limits: (a) mixed EPTB tissue/aspirate specimens, meta-analysis and multicenter validation [3,22]; (b,c) culture against composite clinical/microbiological reference standards in TBM and EPTB cohorts [3,4,7,23,24]; (d) reference-standard-dependent: ≈42% against composite clinical reference standard [3]; 56–63% in pooled analyses [26]; 81–85% against definite/probable TBM definitions [25]; (e) uniform clinical case definition and composite microbiological reference standards, prospective pediatric/adult TBM studies [23,24,27]; (f) pooled, lymph-node studies including a high-HIV setting [3,20]; (g) pleural-fluid estimates differ by assay and reference standard: Xpert MTB/RIF ≈19% against a composite clinical reference and ≈50% against culture [3,28]; Xpert Ultra ≈75% against culture (four studies; very low-certainty evidence) [3]; (h) ADA meta-analysis in tuberculous pleurisy (63 studies; sensitivity 0.92, specificity 0.90) [29]; (i) multicenter adult TBM, composite reference standard; single-study finding [30]; (j,k) restricted to definite TBM; near-100% specificities are study-level findings, not general test characteristics [6,31,32]; (l,m) single-study estimates, abscess/tissue and lymph-node specimens [7,8]; (n) RISK6 evidence derives from pulmonary-TB cohorts [9,10]. AFB, acid-fast bacilli; ADA, adenosine deaminase; AUC, area under the curve; CSF, cerebrospinal fluid; ddPCR, droplet digital PCR; IGRA, interferon-γ release assay; IFN-γ, interferon-γ; mNGS, metagenomic next-generation sequencing; TBM, tuberculous meningitis.

**Table 2:** Site-specific diagnostic algorithms for extrapulmonary tuberculosis. Steps are labeled (G) = current WHO guidance, (S) = supported by site-specific clinical studies, (P) = authors’ proposal pending prospective validation; mNGS-based reflex positioning is a proposal supported by observational accuracy studies, not a guideline recommendation.

Site	First-Line Tests	Reflex (Negative First Line, High Suspicion)	Key Caveats	Evidence
Lymph node	FNA: Xpert Ultra + cytology; excisional biopsy if inconclusive	mNGS on tissue; NAAT for NTM/fungi	Ultra preferred over Xpert; post-treatment sensitivity drops for culture	(G)/(S) first line; (P) mNGS reflex
Osteoarticular	Image-guided biopsy of cellular rim/abscess–tissue interface: histology + Ultra + culture	mNGS (matched/above culture yield)	Preferentially sample viable-appearing tissue; rim/core model extrapolated from pulmonary/animal data (Section 2.1); native specimen mandatory	(S) sampling and mNGS yield; (P) reflex positioning
Pleural	Fluid Ultra + ADA/IFN-γ; closed pleural biopsy	Thoracoscopic biopsy (histology + NAAT + culture)	No single test suffices; combine fluid biomarker + tissue	(G)/(S)
CNS	Centrifuged large-volume CSF: Ultra; uniform case definition	mNGS; ddPCR; repeat LP	Treat probable/definite TBM without awaiting culture	(G) Ultra anchor and case definition; (P) mNGS/ddPCR reflex
Disseminated	Multi-site sampling; mycobacterial blood culture; Ultra per site	Circulating MTB cfDNA (research-stage)	Highest diagnostic uncertainty; composite evidence	(S); cfDNA research
Pediatric/PLWH	Lower threshold to biopsy; gastric aspirate/stool NAAT (detect mainly concomitant pulmonary—often subclinical—disease; adjunctive role rather than site-specific diagnosis); urine LAM (PLWH)	mNGS; Ultra on all obtainable specimens	Lower sensitivities across modalities; faster progression	(G)/(S); stool/gastric-aspirate role adjunctive

ADA, adenosine deaminase; cfDNA, cell-free DNA; CSF, cerebrospinal fluid; FNA, fine-needle aspiration; LAM, lipoarabinomannan; LP, lumbar puncture; NAAT, nucleic acid amplification test; NTM, non-tuberculous mycobacteria; PLWH, people living with HIV; Ultra, Xpert MTB/RIF Ultra.

**Table 3:** Principal gaps in current guidance and corresponding research priorities.

Domain	Current Guidance	Identified Gap	Priority Research Action
Sequencing-based tests	Xpert/Ultra endorsed for most EPTB specimen types; targeted NGS endorsed for drug-resistance detection; no WHO recommendation for mNGS or broad-range tNGS as EPTB diagnostic tests	Standardized accuracy data absent for mNGS/broad-range tNGS in EPTB	Prospective CSF/pleura/bone accuracy studies vs. clinical reference standards
Host biomarkers	No endorsed triage test for EPTB	Pulmonary-centric validation	EPTB-specific transcriptomic/soluble biomarker cohorts
Ultra trace calls	Category defined; interpretation local	Rifampicin-resistance calls indeterminate at trace level	Harmonized reporting rules; outcome-linked trace cohorts
CRISPR/cfDNA	Proof-of-concept only	No reference-standard-compliant prospective data	Multicenter diagnostic accuracy trials in high-burden settings
Algorithm impact	Accuracy studies dominate	Clinical- and cost-impact evidence scarce	Implementation trials with time-to-treatment and sequelae endpoints

cfDNA, cell-free DNA; CSF, cerebrospinal fluid; Ultra, Xpert MTB/RIF Ultra; WHO, World Health Organization.

## Data Availability

No new data were created or analyzed in this study. Data sharing is not applicable to this article.
